# The Selectivity of Polymers Imprinted with Amines

**DOI:** 10.3390/molecules23061298

**Published:** 2018-05-29

**Authors:** Zsanett Dorkó, Anett Nagy-Szakolczai, Blanka Tóth, George Horvai

**Affiliations:** 1 Department of Inorganic and Analytical Chemistry, Budapest University of Technology and Economics, Szent Gellert ter 4., H-1111 Budapest, Hungary; dorko.zsanett@gmail.com (Z.D.); nagyszakolczai.anett@gmail.com (A.N.-S.); 2 MTA-BME Research Group of Technical Analytical Chemistry, Szent Gellert ter 4., H-1111 Budapest, Hungary

**Keywords:** molecular imprinting, selectivity ladder, adsorption, beta blocker, medium effect

## Abstract

One of the main reasons for making molecularly imprinted polymers (MIPs) has been that MIPs interact selectively with a specific target compound. This claim is investigated here with the example of a widely used type of noncovalent MIP, the MIP for the beta blocker propranolol. Adsorption isotherms of this MIP and of a nonimprinted control polymer (NIP), respectively, have been measured with a series of compounds in the porogen solvent acetonitrile. The results, visualized as “selectivity ladders”, show that the MIP binds propranolol and many other amines better than the NIP does, but the selectivity of the MIP is actually inferior to that of the NIP. The selectivity of either polymer for propranolol is modest against many amines, but is remarkable with respect to other compounds. The contribution of imprinting towards selectivity can be better appreciated when three MIPs, made with different amine templates, are compared among themselves. Each MIP is seen to bind its own template slightly better than the other two MIPs do. In media different from the porogen, the selectivity patterns may change substantially. Propranolol seems to have properties that make it stand high on the selectivity scale in different solvents, albeit for different reasons.

## 1. Introduction

Molecular imprinting is an intensively researched field of chemistry. For quite a few years, the annual production of papers dealing with molecularly imprinted polymers (MIPs) has been around one thousand [1]. There have been many possible applications described for MIPs [2,3,4,5,6,7,8,9,10] and virtually all of these proposals claim to utilize the unique selectivity patterns of MIPs. As we have recently shown, Ref. [11], claims about MIP selectivity need to be carefully stated. It was found that an MIP imprinted noncovalently with the beta blocker compound propranolol could bind only about five times as much propranolol as dibenzylamine (DBA) under identical conditions. The experiments were done in the porogenic solvent of the MIP, which is the medium held best for rebinding purposes [12,13,14,15,16,17]. Dibenzylamine is a secondary amine like propranolol, but otherwise its structure and basicity are very different from those of propranolol. In the same work a compound somewhat more similar to propranolol ((*R*)-(−)-2-benzylamino-1-phenylethanol, RBz) was even less differentiated by the MIP. On the other hand, binding selectivity for propranolol against nonrelated compounds like some acids, amides or terbutylazine were excellent. These results confirmed earlier observations that MIP selectivity against close analogs of the template may be low [18,19], but the selectivity against many other compounds can be excellent, although occasionally compounds not obviously related to the template may be strongly bound by the MIP [19,20].

The present work was initiated with the goal to better understand the selectivity patterns of MIPs. The first part of the paper shows the difficulties of adequately defining the meaning of MIP selectivity. After this, a proposal is made for a simple, practical and thermodynamically based definition of MIP selectivity. Using this definition and a concomitant simple graphical presentation method, the selectivity pattern of the widely studied propranolol MIP in its porogenic solvent is shown, and it is compared with the selectivity pattern of the corresponding nonimprinted control polymer (NIP), and also with the selectivity patterns of MIPs imprinted with other amines. Changes in the propranolol MIP’s selectivity pattern when the rebinding medium is varied are also presented. Finally, the practical consequences of this study are derived.

## 2. Theory

### 2.1. Definitions of MIP Selectivity

The ultimate goal of molecular imprinting is to obtain an imprinted polymer with some practically useful features. Very often, this feature is the separation of a compound (or of a group of compounds) from a matrix (e.g., by solid phase extraction, chromatography, binding assay, etc.), or the determination of a compound by selective interaction with an MIP sensor. In virtually every application, the MIP is expected to demonstrate some sort of selectivity for the target compound(s) against other compounds in the matrix. The achieved level of selectivity needs to be proven in the real life application. These applications being rather varied, the methods of verifying MIP selectivity are also manifold. For example, if the MIP is used for solid phase extraction (SPE or MISPE), the recovery of the analyte is shown to be high, whereas the recovery of certain matrix components is shown to be low. In chromatography on MIP columns, the ratio of the respective retention factors of the target compound and of an interferent is often used to demonstrate the achieved selectivity. In binding assays, the ratio of IC50 values (IC50: the concentration of the studied compound which displaces 50% of a bound tracer) is used to quantify selectivity. The selectivity of an MIP sensor may be characterized by the small magnitude of the effect on the sensor signal, caused by the presence of an interferent in the sample containing the target compound.

Such practical demonstrations of MIP selectivity, while justified in their own place, have several drawbacks. Firstly, most of them provide information that is only valid in the exact experiment done, but there may be no way to prove that, in different sample matrices, at different target compound concentrations and at different interferent concentrations, the selectivity will still be the same or sufficient. For example, we have shown [21] that chromatographic separation factors on MIP columns are concentration dependent. Moreover, quite unexpectedly, they depend also on the length and diameter of the column [22] and therefore the separation factor measured with one column is not transferable to another column of different dimensions.

The second problem with application oriented selectivity characterization is that selectivity values obtained for one application are usually not transferable to other applications. For example, IC50 values obtained in a binding assay are not directly comparable with SPE recoveries, even if a rough qualitative relationship may exist.

The selectivity requirements of different methods may also differ substantially. In chromatography, minor differences of retention (e.g., separation factor of 1.5) may be sufficient for perfect separation, whereas, with a sensor, one may require that its analyte-to-interferent sensitivity ratio be about 100. Thus, the same MIP may be called very selective in one application, and almost non-selective in another.

The problem of defining and measuring selectivity is actually not unique to molecular imprinting. The International Union of Pure and Applied Chemistry (IUPAC) has published two recommendations [23,24] about selectivity in analytical chemistry, and none of these has offered a quantitative measure of the selectivity of analytical methods. In view of this, one cannot expect to find a general solution to the problem of characterizing MIP selectivity, but it appears to be meaningful to search for some better methods than the ones cited above.

### 2.2. The Selectivity Ladder: A Simple Method to Show MIP Selectivity Patterns

We have shown recently [11] that the adsorption isotherms of MIPs can conveniently be compared in log *c*-log *q* plots, i.e., in figures where the logarithm of the equilibrium adsorbed concentration of an investigated compound on an MIP (or on a NIP) is plotted as a function of the compound’s logarithmic equilibrium solution concentration. One may plot and compare in the same diagram the isotherms of several compounds on the same MIP, or the isotherms of a single compound on an MIP and its NIP, respectively, or the isotherms of the same compound on two different MIPs. Like in many other publications, we have also found [11] that such log *c*-log *q* plots are often linear in a quite wide range of the respective concentrations. (Note that this linearity means that the system observes the Freundlich equation, but, in the present context, there is no attempt to interpret the isotherms with any model.) We have also shown [11] that the vertical distance between the isotherms may be interpreted as the logarithm of the selectivity (when the adsorption of two compounds is studied on the same polymer) or as the logarithm of the imprinting factor (when the adsorption of the template is plotted on the MIP and the NIP, respectively). This interpretation of the imprinting factor is very close to that of Lanza and Sellergren [25].

**Figure 1:**
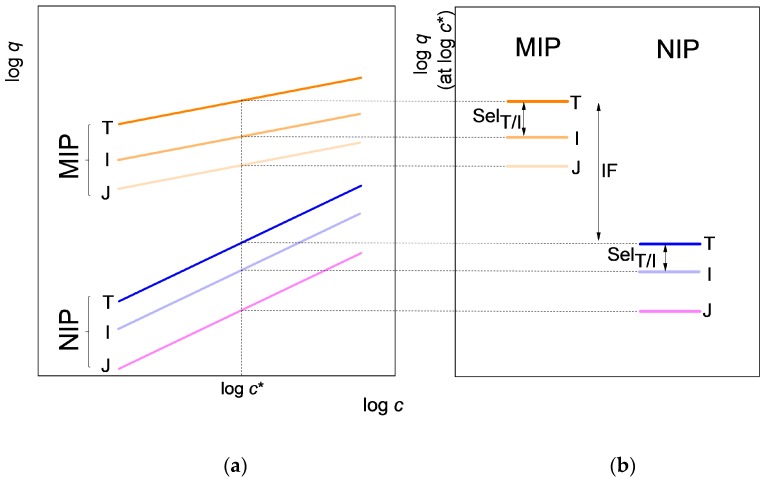
(**a**) Schematic isotherms of three compounds on an imprinted polymer (MIP) and on its control polymer (NIP). The template of the MIP is T, while I and J are compounds related to T; (**b**) the selectivity ladders of the MIP and the NIP, respectively, as derived from the isotherms. Arrows show the imprinting factor (IF) and the selectivity for the template T against compound I (Sel_T/I_), respectively. Note that the selectivity of the MIP and of the NIP, respectively, are different.

By looking at Figures 4 and 5 of our earlier paper [11], one may additionally observe that the log *c*-log *q* adsorption isotherms of different amines on the propranolol MIP are not only straight lines, but they are also approximately parallel with each other. The isotherms of the same amines on the NIP are also approximately parallel, albeit with a different slope, than on the MIP. This situation is schematically shown here in Figure 1, with respect to three different adsorbed compounds, one being the template, T, and the two others, denoted by I and J, respectively, some compounds more or less closely related to the template. As noted above, the selectivities and the imprinting factor can be characterized by the vertical distances between the corresponding straight lines, as shown in Figure 1 by arrows. Since such distances are more or less independent of the concentration if the two isotherm lines are approximately parallel, it is sufficient to read the respective log *q* values from all isotherms at a single, conveniently selected concentration, c*, or more correctly at log c*. To easily appreciate selectivities and imprinting factor (IF) values, one may plot the respective log *q* values at log c* in a separate diagram, as shown in Figure 1b The advantage of this plot is that one can show in the same plot in a very simple way the relative position of the isotherms of many compounds, even on several polymers and in several solution media. Such “selectivity ladders” have been often used in electrochemistry to show the relative position of standard potentials. The differences between standard potentials have also been interpreted as selectivities, e.g., in ion selective electrode potentiometry [26].

An alternative possibility for evaluating adsorption isotherms consists of fitting some isotherm equation to the measured data, and comparing the constants of the equations between different adsorbed compounds or between different polymers. We have demonstrated recently that the necessary conditions for the accurate determination and for the correct interpretation of these constants are rarely satisfied by isotherm measurements on MIPs [27]. The selectivity ladder method presented here requires much less and also less demanding experimentation.

The selectivity ladders will be used here to make conclusions about MIPs by comparing the equilibrium adsorption of many compounds on a variety of MIPs and on their NIP.

## 3. Results and Discussion

### 3.1. Comparison between the MIP and Its NIP

**Figure 2:**
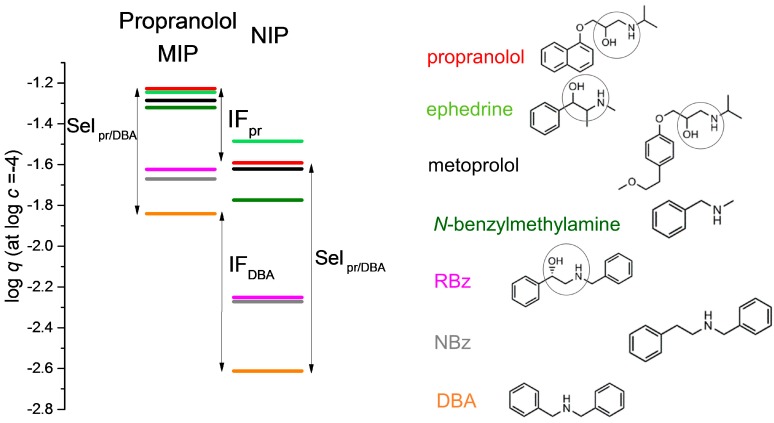
The selectivity ladder of seven amines on the propranolol MIP and its NIP, respectively, measured in the porogen acetonitrile. The structure of the compounds is shown on the right-hand side, with their names following the color coding of the plot.

Figure 2 shows the selectivity ladders, i.e., the respective log *q* values for seven different compounds on the propranolol MIP and the corresponding NIP, calculated at log *c* = 10^−4^ M from the equilibrium measurement data, using the respective adsorption isotherms, or from single point measurements with extrapolation on a short distance. Figure 2 also shows the structures of the seven investigated compounds using the same color coding as on the selectivity ladders. Metoprolol, ephedrine and RBz contain the same aminoethanol group as propranolol (see circles on their structures in Figure 2). These three compounds may be considered as close analogs of propranolol in terms of their structures. The other three amines in Figure 2 are secondary amines like propranolol, but otherwise not very similar to it. In these three amines, there is at least one phenyl ring in benzylic position to the amino group, unlike in propranolol, where the amino group is within an aliphatic chain.

One can make several observations by inspecting Figure 2. Every individual compound is better adsorbed by the MIP than by the NIP. For the template propranolol, the distance between its respective adsorption levels on these two polymers gives the logarithm of its imprinting factor (shown by the two-headed arrow IF_pr_). The distance between the respective lines of two amines on the same polymer may be interpreted as the logarithmic measure of selectivity of the polymer for the amine in the higher position against the one in lower position. The two two-headed arrows Sel_pr/DBA_ show the selectivity of the MIP and of the NIP, respectively, for propranolol against dibenzylamine. 

What immediately strikes the eye is that the selectivity range of the propranolol MIP for all investigated amines, similar or dissimilar to propranolol, is only about 0.7 logarithmic units (from the line of propranolol to the line of dibenzylamine), which is equivalent to an at most five times higher binding (*q*) of propranolol than of any other compound at the same solution concentration (10^−4^ M)). This means that the selectivity of the propranolol MIP even against amines “unrelated” to propranolol is quite modest. Note that, at the same time, the distribution coefficients of the investigated compounds are very high. The log *q* values are between −1.2 and −1.9 at the log *c* value of −4.0 (Figure 2), i.e., even the lowest distribution coefficient, that of dibenzylamine, is higher than 100. Thus, the possibility of using a large span of useful distribution coefficients (from, say, 1, to several hundred) is not utilized efficiently. The selectivity against the three closer analogs (aminoethanols) is really small, less than 0.1 logarithmic unit. One should note here that the selectivity of the propranolol MIP against many compounds without an amino group is very high, but this is also true for the NIP [11].

Surprisingly, the selectivity range of the NIP is broader than that of the MIP because it is more than 1.1 logarithmic unit. In other words, the NIP is more selective for propranolol against some amines than the propranolol imprinted MIP. This is just the opposite of what one would expect, i.e., that imprinting should improve the selectivity. Actually, one can see selectivity improvement against a single compound, ephedrine, in Figure 2, but not against any other compound. A further surprising consequence of these results is that the apparent imprinting factor for compounds like dibenzylamine (two-headed arrow IF_DBA_ in Figure 2) is much higher than for the actual template, propranolol (two-headed arrow IF_pr_).

There are some interesting consequences of these results for the evaluation of novel MIPs. It has become a commonplace in the MIP literature to compare the binding of the template on the MIP with its binding by the NIP. When the MIP binds significantly more template than the NIP, this is considered as the proof that imprinting was successful because it is thought that the MIP contains some additional strong and selective imprinted sites that cannot be found on the NIP. The observation made here, that the MIP is not necessarily more selective for the template than the NIP, seems to contradict this line of reasoning.

### 3.2. Comparison between MIPs of Different Templates

**Figure 3:**
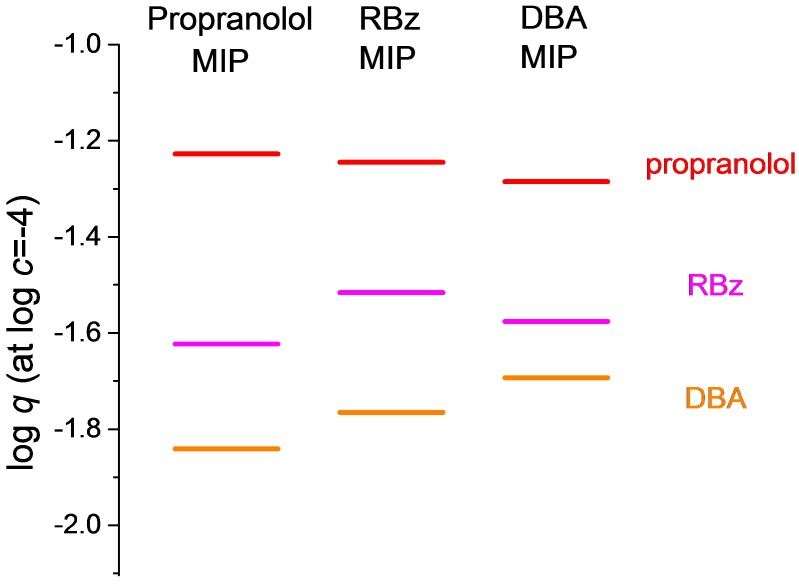
The effect of imprinting on three different amine MIPs. Adsorption of each compound was measured on all three polymers. All solutions were made in acetonitrile.

If one would like to study the effect of imprinting on selectivity, it seems to be more fruitful to study a series of MIPs imprinted with different templates than to compare a single MIP with its NIP. Figure 3 shows the respective selectivity ladders of three amines (propranolol, RBz and dibenzylamine) on the three MIPs imprinted by these compounds. The data have been calculated from single point adsorption experiments (1 mL of 0.5 mM solution added to 10 mg polymer) by extrapolation on a short distance as described above.

One can see in Figure 3 that, irrespective of the template used, all three polymers preferentially bind propranolol, while the binding of RBz is second and of dibenzylamine is third. On the other hand, if one compares the binding of any of the three amines on the three MIPs, one can observe that the particular amine is more strongly bound on its own MIP than on the other two MIPs. The differences are small in any case.

These results show that imprinting indeed imparts a certain selectivity improvement to the MIP with respect to its own template. This improvement may be, however, modest, because imprinting with the template increases also the binding of the other amines. Since some of these amines may be inherently preferred by the functional monomers, as for instance propranolol or ephedrine are apparently preferred by methacrylic acid (Figure 2, NIP data), the effect of imprinting with other amines may not be sufficient to overcome the inherent preference of the monomer for these compounds. 

The inherent preference of common acidic functional monomers for propranolol and its analogs may be one reason for the popularity of these compounds as templates in noncovalent imprinting studies. The other side of this remark is that imprinted polymers with remarkable selectivity for propranolol (in acetonitrile) can be made by using other amines than propranolol as the template. Indeed, as Figure 3 shows, the MIPs imprinted with RBz or DBA might be used as adsorbents selective for propranolol. Such replacement of the target compound by one of its analogs as the template is well known in molecular imprinting, but it may not be a commonplace that, in such cases, the binding of the alternative template by the MIP can be less than that of the target compound.

### 3.3. Comparison between Different Rebinding Media

The medium chosen for making the MIP by polymerization may differ from the medium of its intended practical application. For technical reasons, the polymerization is often made in aprotic media, while, in the applications, protic solvents may be preferable or necessary. Applications in aqueous media are obvious examples. In liquid chromatography on MIP columns, it is common to use acetonitrile with up to a few percent acetic acid as the eluent. One reason for using acetic acid in the eluent is that otherwise the retention of the compounds to be separated would be impractically high.

**Figure 4:**
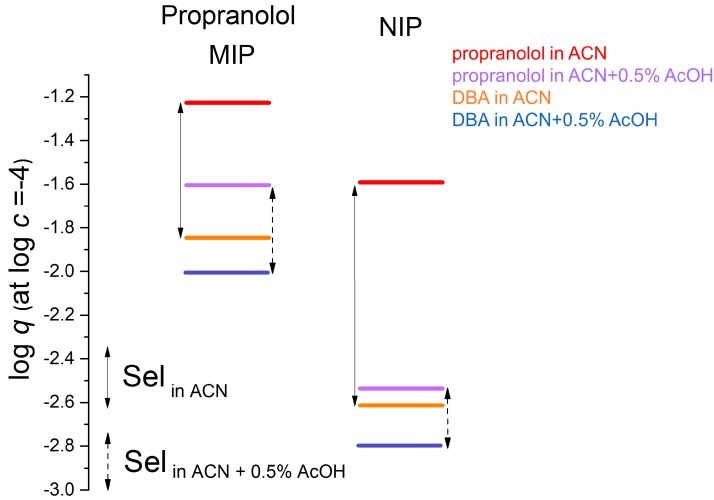
Propranolol and DBA adsorption on propranolol MIP and NIP, respectively, in acetonitrile (ACN) and in ACN + 0.5% acetic acid (AcOH).

Figure 4 uses adsorption data measured [11] with the propranolol MIP and its NIP in acetonitrile, and also in acetonitrile containing 0.5% acetic acid (AcOH). Two compounds have been investigated, propranolol and dibenzylamine. The addition of acetic acid decreased the adsorption of both compounds, but not in equal measure. The binding of propranolol was reduced more, and therefore the selectivity for propranolol is less in the acidic medium, as shown by the two two-headed arrows in the MIP ladder. The effect of acetic acid on the NIP is qualitatively the same as on the MIP, but quantitatively the drop in propranolol binding due to the acid is more drastic. Thus, the selectivity of the NIP for propranolol is rather meagre in the acidic medium.

As noted, Figure 4 shows that the selectivity of the MIP in the acidic medium is less than in pure acetonitrile (ACN). However, the imprinting factor for propranolol is much higher in the acidic medium. This is so because template adsorption has been differently affected on the MIP and on the NIP by the change of medium. Imprinting factors are sometimes determined by elution chromatography, using columns made from the two polymers. The eluent is typically not the pure porogen, and therefore the observed imprinting factor may not adequately reflect the imprinting effect that occurred in the porogen.

The propranolol MIP, which has been prepared in acetonitrile, may be also investigated in a very different solvent, toluene. It has been found (data not shown here) that the selectivity of this MIP for propranolol against dibenzylamine is slightly better in toluene than in acetonitrile. This experiment shows that the selectivity of an MIP for its template may be higher in an alternative solvent than in the porogen.

In buffered aqueous media, the selectivity may be strongly influenced by the hydrophobicity of the compounds. Andersson [28] had worked with essentially the same propranolol MIP composition as used in the present paper. He found that, in a radiotracer binding assay, carried out in aqueous medium, the MIP was remarkably selective for propranolol against three other beta blockers, metoprolol, timolol and atenolol. This is surprising because all three compounds share the same side chain as one can find in propranolol (attached to the naphtyl ring, see Figure 2). However, as seen above, metoprolol is hardly differentiated from propranolol by the propranolol MIP in the porogen acetonitrile. One should note, however, that the lipophilicity of propranolol is much higher than of the other three compounds. Propranolol’s log *P* is 3.48, while the other three compounds are between 0.16 and 1.88 [29]. Thus, the observed high selectivity for propranolol in aqueous media may be due, at least in part, to its higher lipophilicity. Clearly, there must be also an imprinted part of the selectivity because the MIP showed preference for the template *S*-propranolol against *R*-propranolol in the aqueous buffer.

One can see from these results that the effect of the rebinding medium on MIP selectivity can be very complex. Thus, for theoretical work and for generic characterization of an MIP, one should prefer making equilibrium adsorption measurements in the porogen. For practical applications, however, it is better to test the MIP in the application itself, by doing a sufficient number of experiments to cover all the likely future conditions of the application. Extrapolation from a few experiments to other conditions may be risky.

## 4. Materials and Methods

### 4.1. Materials

Methacrylic acid (MAA), ethylene glycol dimethacrylate (EDMA), propranolol hydrochloride, dibenzylamine (DBA), (*R*)-(−)-2-benzylamino-1-phenylethanol (RBz), *N*-Benzyl-2-phenethylamine (NBz), metoprolol tartrate, ephedrine, acetic acid (AcOH) were purchased from Sigma (St. Louis, MO, USA), *N*-benzylmethylamine from Alfa Aesar (Haverhill, MA, USA), azobisisobutyronitrile (AIBN) from Fluka (Buchs, Switzerland), HCl from Riedel-De Haën (Seelze, Germany), acetonitrile and methanol from Merck (Darmstadt, Germany) Water was purified with a Milli Q Direct 8 system (Millipore, Burlington, MA, USA).

Prior to use, propranolol hydrochloride and metoprolol tartrate were transformed to their free base form, by means of neutralization with 0.2 M NaOH solution followed by extraction with methyl *tert*-butyl ether.

### 4.2. Instrumentation

The following instruments were used: PTR-35 multirotator (Grant-bio, Cambridge, GB), Minispin centrifuge (Eppendorf, Hamburg, Germany), Series 200 HPLC (Perkin Elmer, Waltham, MA, USA), Purospher RP18-e (125 × 3 mm, 5 μm, Merck) reversed phase column.

### 4.3. Polymer Preparation

Polymers were prepared as described previously [11], using the method of Andersson [28] for the propranolol MIP. The nonimprinted polymer (NIP) was also prepared in the same way but omitting the template.

The template (propranolol or DBA or RBz), functional monomer (MAA), crosslinker (EDMA), initiator (AIBN) and the polymerization solvent (ACN) were mixed in a glass vial. After purging with argon for 10 min, the vial was sealed and the mixture was polymerized under a UV source (366 nm) for 24 h at room temperature. The formed bulk polymers were crushed and ground in a mortar. The NIP was cleaned using methanol. The template was removed from the MIPs by washing several times with 0.01 M HCl solution in methanol-water 1:1, and then with methanol. After washing, the polymers were dried overnight.

The molar ratio of template:functional monomer:crosslinker was 1:8:40. The solvent/total monomer volume ratio was 1.3 and the amount of initiator was 1.3 mole% of the total monomer amount.

### 4.4. Equilibrium Binding Experiments

Equilibrium binding experiments were carried out at room temperature (25 ± 2 °C) in polypropylene microtubes. The adsorption isotherms were measured by varying the initial concentration and the volume of the analyte solutions. In the one-point measurements, the initial concentration of the analyte was chosen such that the final solution concentration should be close to 10^−4^ M. The equilibration was done by rotating the mixture for 30 min; then, the samples were centrifuged. The supernatant was diluted with the HPLC eluent and was injected into the HPLC system to quantify the unbound analyte concentration. The HPLC measurements were carried out using a Purospher RP18-e (125 × 3 mm, 5 μm, Merck) reversed phase column. The eluent contained phosphate buffer and ACN in different ratios, depending on the compound to be measured (phosphate buffer: 10 mM NaH_2_PO_4_, the pH was adjusted to 3 by H_3_PO_4_). The injection volume was 10 μL, and the flow rate was 0.6 mL/min.

## 5. Conclusions

The main goal of molecular imprinting is to make a polymer that has selective properties towards some target compound(s). This paper has shown among others that:

- It is difficult to define, let alone measure, the selectivity of MIPs, in such a way that the obtained selectivity value could be generally used in all applications of the MIP. In other words, there is no such thing as “the” selectivity of an MIP.
- For fundamental studies of the imprinting effect, it is useful to do equilibrium binding experiments in the porogen. In other media, selectivities may be quite different.
- If the adsorption isotherms of different substances in a particular medium are approximately parallel on the log *c*-log *q* plot, it is useful to study the selectivity ladder. The selectivity ladders of different polymers, or of the same polymer in different media, may also be compared.
- When demonstrating the good selectivity of an MIP against a compound “similar” to the template, one should be aware that great similarity in the structural formulas may hide big differences in some properties (like hydrophobicity), which are relevant for adsorption.
- Imprinting, even if successful in increasing the binding of the template against the NIP, does not necessarily lead to improved selectivity compared to the NIP. Imprinting by a particular compound (the template) will often increase the binding of other compounds as well, eventually more than for the template, thus leading to reduced selectivity.
- The selectivity of MIPs in real life applications needs to be carefully studied. Demonstration of selectivity in a single experiment may be insufficient.

Some of these conclusions will have been drawn by other authors, based on other experiments. In this study, they appear, however, together, all based on a single type of experiment. This experiment is an equilibrium binding measurement, and thus it conveys thermodynamic results, which are generally better defined than selectivity measurements by other methods. 

Baggiani and coworkers had shown some years ago [30] that, for efficient noncovalent imprinting, it is usually necessary that even the NIP does appreciably bind the template. The present work gave some examples that, for achieving substantial selectivity of an MIP for its template, it may often be necessary additionally that the NIP itself also shows some selectivity for that compound.

Strictly speaking, the results of this paper are valid only for the systems studied in this work. With other imprinting systems (e.g., in stoichiometric noncovalent imprinting [31,32]), some of the conclusions made here may not be valid. In any case, this paper may be generalized in the sense that it directs attention to some factors that are important when developing novel MIPs or novel applications for MIPs.

In real life applications, selectivity needs to be effective in the simultaneous presence of the target compound with other compounds. The present study has been based on separate measurements of individual compounds, one at a time. It is possible to do equilibrium adsorption measurements also in mixtures. In some, but not all cases, the selectivity in mixtures may be estimated from separate measurements with the individual compounds [33].
